# Adolescent obesity treatments: news, views, and evidence

**DOI:** 10.20945/2359-3997000000393

**Published:** 2021-09-29

**Authors:** Louise Cominato, Ruth Franco, Durval Damiani

**Affiliations:** 1 Universidade de São Paulo Unidade de Endocrinologia Pediátrica São Paulo Brasil Unidade de Endocrinologia Pediátrica, Universidade de São Paulo, São Paulo, Brasil

**Keywords:** Obesity, overweight, adolescent, treatment, liraglutide

## Abstract

Obesity is a complex and multifactorial disease that is influenced by physiological, environmental, socioeconomic, and genetic factors. In recent decades, this serious disease has impacted a large number of adolescents as a result of lifestyle factors. A lack of exercise and the consumption of excessive calories from an inadequate diet are the main contributors to adolescent obesity. However, genetic and hormonal factors might also play a role. The short- and long-term consequences of this disease include chronic issues such as type 2 diabetes and cardiovascular disorders and an increase in early mortality rates. Although it is a serious disease, obesity in adolescents can be controlled with diet and exercise. When these lifestyle changes do not obtain the expected results, we can intensify the treatment by adding medication to the practice of diet and exercise. Additionally, for more severe cases, bariatric surgery can be an option. The purpose of this review is to clarify the current epidemiology, risks, and comorbidities and discuss news about the main treatments and the necessary improvements in this context.

## INTRODUCTION

### Current prevalence and future risks of obesity

The World Health Organization (WHO) defines obesity as “abnormal or excessive fat accumulation that presents a risk to the health” ( 1 ). Furthermore, in adults, obesity is characterized as a body mass index (BMI) equal to or above 30 kg/m^2^ and is subclassified into class I (30-34.9 kg/m^2^), class II (35-39.9 kg/m^2^), and class III (≥40 kg/m^2^) ( 2 ). In adolescents (10-19 yrs) worldwide, obesity is a public health problem that affects low-, medium-, and high-income countries and is more frequent in urban centers where access to high-calorie foods is easy and common ( 1 ). According to the WHO, obesity in adolescents and in children (≥5 yrs) is defined as BMI>+2SDS and severe obesity as BMI>+3SDS. In children (≤5 yrs), obesity is defined as BMI>+3 SDS, overweight as BMI SDS between +2 and +3, and risk for overweight between +1 and +2. If we use percentiles, obesity is defined as BMI over the 95^th^ percentile, while overweight is defined as BMI between percentiles 85 and 95 ( 1 ).

Obesity is a complex, incompletely understood, serious, chronic disease that is part of a cluster of noncommunicable diseases that can be avoided or treated, and yet, over 340 million children and adolescents were overweight or with obesity in 2016 ( 1 ). In 2008, a Brazilian study identified that 20.5% of adolescents were overweight and 4.9% were with obesity, and these numbers were probably due to the observation that these adolescents ingested high amounts of calories from unhealthy sources. Additionally, obesity was more common in male adolescents, and the consumption of industrialized and high-fat foods was higher among adolescents than among adults and the elderly ( 3 ). In 2015, when evaluating the risks of developing cardiovascular diseases (CVDs) in 73,399 students, another Brazilian study showed that 17.1% of students were overweight, while 8.4% were with obesity. Overweight was more prevalent in females (12-14 yrs), while obesity was more common in males ( 4 ). More recent data from the Food and Nutrition Surveillance System (IBGE – Brazilian Institute of Geography and Statistics) showed that 16.33% of Brazilian children (5-10 yrs) were overweight; 9.38% were with obesity, and 5.22% were with severe obesity. Regarding adolescents, 18% were overweight; 9.53% were with obesity, and 3.98% were with severe obesity ( 5 ).

Theoretically, obesity occurs when energy intake exceeds consumption. In practice, obesity is a complex multifactorial disease with physiological, environmental, socioeconomic, and genetic influences. The exact association among the multiple factors related to the onset of obesity is poorly understood, but it has already been shown that the risks for obesity begin in the prenatal period. Maternal obesity, excessive gestational weight gain, and smoking are well-established risk factors for obesity (( 6 , 7 ). In the postnatal period, early weight gain increases the risk of adolescent obesity and CVD ( 7 ), while breastfeeding decreases the risk in a dose-dependent manner ( 8 ).

Obesity is also related to behavioral factors. The diet quality, exercise habits, and psychosocial status can be crucial in the development of obesity. However, a predisposition to obesity exists, and the disease is not exclusively behavioral. Many patients with obesity have enormous difficulty losing and maintaining weight loss. The annual chance of a patient with obesity to reach the ideal weight was 1 in 210 for men and 1 in 124 for women. In addition, restricting these data to the population with morbid obesity (BMI, 40-44.9 kg/m^2^), this probability decreases to 1 in 1290 for men and 1 in 677 for women ( 9 ).

Genetically, obesity can be syndromic, monogenic, oligogenic, or polygenic. Monogenic and syndromic obesity is rare and occurs when a single gene mutation could result in severe obesity, irrespective of environmental stimuli. The main changes involved in monogenic nonsyndromic obesity occur in the leptin-melanocortin pathways ( 10 ). Mutations in *LEP* and *LEPR* trigger rapid weight gain, behavioral problems when food is denied, hyperphagia, hypogonadotropic hypogonadism, defective T-cell mediated immunity and low blood pressure ( 10 ). Additionally, mutations in the *MC4R* gene may be responsible for 6% of severe pediatric obesity cases and are associated with accelerated growth and hyperinsulinemia ( 11 ). On the other hand, environmental factors can exacerbate the progression of oligogenic and polygenic obesity. This genetic background is more common in patients with a genetic predisposition to weight gain. The list of polygenic loci associated with obesity and body fat distribution traits increases every day as research technology advances ( 10 ). A genome-wide association study (GWAS) quantified the relationship between each of the 2.1 million common genetic variants and BMI in over 300,000 individuals, but none of the individual variants were responsible for a large proportion of obesity cases. The strongest association was observed for a common variant at the fat-mass-and-obesity-associated gene ( *FTO* ) locus (on 16p11.2) ( 12 ). Obesity-associated sequences within *FTO* appeared functionally connected through a noncoding ribonucleic acid (ncRNA) to increase the expression of *IRX3* , an adipose tissue gene that has been shown to have the effect of browning white fat. Furthermore, the allele linked to the risk of obesity was associated with a statistically robust but clinically modest increase in weight of approximately 1 kg per inherited risk allele ( 12 ). Together, the behavioral and genetic factors emphasize the importance of every adolescent with severe obesity undergoing a clinical and laboratory workup searching for developmental delays and possibly metabolic syndrome, neurological disorders, endocrinopathies, or monogenic defects ( 13 ).

Obesity can trigger a series of long-term comorbidities, and adolescents are too young to understand the future consequences of their current choices. An estimate showed that 57.3% of children today will be adults with obesity at 35 yrs, and children who are currently with obesity have a 6.1% chance of not being with obesity at age 35 ( 14 ). In addition, 6% of children with normal weight, 29% of overweight children, 56% of children with obesity, and 80% of children with severe obesity will grow up to be adults with class II or III obesity ( 15 ).

### Comorbidities associated with adolescent obesity

**Figure 1:**
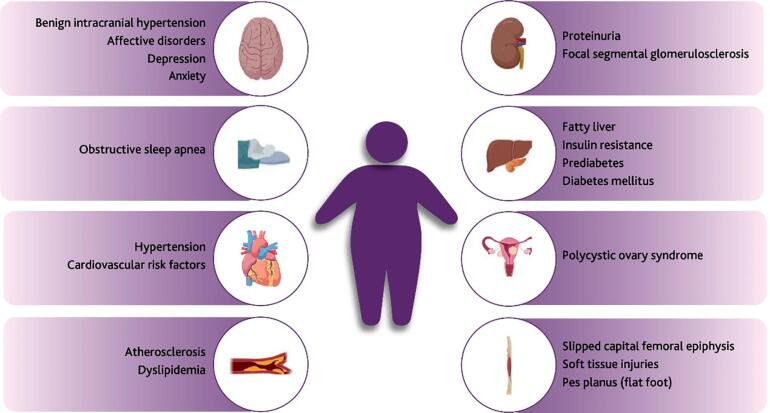
Obesity-associated comorbidities. Created with BioRender.com ( 1 , 2 , 17 ).

The main comorbidities associated with adolescent obesity are described in Figure 1 ( 1 , 2 , 16 ). CVD, type 2 *diabetes mellitus* (DM2), musculoskeletal disorders, and certain types of cancer (endometrial, breast, and colon) are the main long-term consequences of obesity ( 1 , 16 ). Early obesity can increase the risk of death from CVD or any other cause in adulthood and decrease the life expectancy that would otherwise be achieved ( 17 ).

Height and weight data for 2.3 million adolescents were measured, and the risk of death from coronary heart disease (hazard ratio, 1.5; 95% confidence interval [CI], 1.3 to 1.8) and death from total cardiovascular causes (hazard ratio, 1.3; 95% CI, 1.2 to 1.5) was significantly higher among adolescents with BMI values in the 50th to 74th percentiles than among those in the 5th to 24th percentiles. Furthermore, adolescents who were overweight (85th to 94th percentiles) had hazard ratios of 3.0 (95% CI, 2.5 to 3.7) for death from coronary heart disease, 1.8 (95% CI, 1.3 to 2.5) for death from stroke, 1.5 (95% CI, 1.1 to 1.9) for sudden death, and 2.2 (95% CI, 1.9 to 2.6) for death from total cardiovascular causes ( 17 ).

Quantitative T2 neuroimaging and functional magnetic resonance imaging showed that obesity in children is associated with structural abnormalities in the hypothalamus. These abnormalities, probably caused by the inflammatory process resulting from obesity, can potentially compromise the center for the regulation of appetite. Children with obesity had longer T2 relaxation times, which is consistent with the nonspecific reactive change in glial cells in response to damage to the central nervous system (gliosis). These abnormalities are reflected in adults with alterations in the hypothalamic functional response to glucose ( 18 ).

### Modalities of treatment and their caveats

Adolescent obesity can be treated with lifestyle changes, pharmacotherapy, and surgery ( 19 ). All these interventions, when properly applied, have exhibited positive results in the treatment of obesity ( 19 ). A systematic review showed that all types of interventions have positive and negative points, but the surgical interventions resulted in the largest BMI reduction (moderate quality of evidence) ( 19 ). Lifestyle change strategies should be the primary goal for the prevention or treatment of pediatric obesity ( 20 ).

### Difficulties of lifestyle change

Preventing obesity in adolescence is always the best choice. However, when this disease is already established, the better strategy is a combination of diet and exercise in the short to medium term. Furthermore, the lifestyle change strategy must be maintained in association with any additional therapy chosen, including pharmacotherapy or bariatric surgery ( 21 , 22 ).

Compared with no-treatment control conditions, lifestyle interventions resulted in significant weight loss in all studies included in a systematic meta-analysis review. Cardiometabolic outcomes such as low-density lipoprotein cholesterol, triglycerides, fasting insulin, and blood pressure were also significantly improved ( 23 ). However, adapting to a new lifestyle is not a simple task, and most of the studies evaluated had a dropout rate ≤ 30% at 6 months or < 40% at 1 yr ( 23 ). Therefore, the main evidence suggests that a multidisciplinary approach is the most accurate and effective strategy to induce children and adolescents to maintain the suggested lifestyle changes and confirms that family involvement and peer support with group meetings are the key to ensuring adherence to a healthier option ( 21 , 23 , 24 ).

### Pharmacotherapy considerations

Lifestyle changes are not always sufficiently effective to produce satisfactory weight loss. Therefore, pharmacotherapy, in addition to behavioral changes, is necessary in these cases ( 25 ). Although the number of anti-obesity medications approved by regulatory agencies for the treatment of obesity in adults has increased, pharmacotherapy options for young people remain limited. In Brazil, liraglutide is approved by the Brazilian regulatory agency – Anvisa – for the treatment of obesity in adolescents and in adults ( 26 ). Orlistat, sibutramine, metformin, fluoxetine and topiramate have been studied and used without labeling to treat pediatric obesity ( 27 ). Table 1 describes the mechanism of action of these drugs, and Table 2 shows a comparison of the efficacy and safety of the pharmacotherapy used in Brazil for the treatment of adolescent obesity. In the USA, liraglutide, orlistat and phentermine (in individuals above 16 yrs and during 3 months maximum) are approved by the local regulatory agency – FDA – to treat adolescents with obesity, while there is no medication approved for this purpose in Europe ( 26 , 28 ).

**Table 1:** Mechanism of action of some medications that can be used to treat adolescent obesity in Brazil

Drug	Mechanism of action
Liraglutide ( 34 )	The GLP-1 receptor is expressed in neurons of the arcuate nucleus of the hypothalamus involved in weight loss. Liraglutide is aGLP-1 receptor agonist that directly stimulates neurons that synthesize POMC/CART (increasing satiety). Indirectly inhibits neurotransmission in neurons that express NPY/AGRP (reducing hunger), GABA-dependent signaling pathways. Liraglutide binds in key areas linked to the control of energy balance, circuits linked to reward and pleasure. Decrease of speed of gastric emptying is temporary. Its action independent of the vagus nerve.
Orlistat ( 31 )	Inhibitor of gastrointestinal lipases that binds to the active site of the enzyme through covalent binding. This binding prevents digestion and intestinal absorption of about one-third of the ingested triglycerides that are eliminated in the stool.
Sibutramine ( 31 )	Blocking the reuptake of norepinephrine and serotonin that triggers neurotransmission modulation and increased feeling of satiety.
Metformin ( 31 )	The exact mechanism of action is not fully understood. Reduces blood glucose due to the insulin-sensitizing effect in liver and muscle tissue. In the hepatocyte, induces gluconeogenesis and glycogenolysis inhibition, and glycogenesis stimulation. In peripheral insulin-dependent tissues, especially in skeletal muscle, glucose uptake increases, causing a rapid reduction in blood glucose.
Topiramate ( 47 )	It acts on several neurotransmitters, having inhibitory effects on glutamate receptors and some types of voltage-gated calcium and sodium channels. It modulates some potassium channels, GABA-A receptors, in addition to being a weak inhibitor of carbonic anhydrase.

GLP-1: glucagon-1-like peptide agonist; POMC/CART: pro-opiomelanocortin and regulatory transcripts by cocaine and amphetamine; NPY/AGRP: neuropeptide Y and agouti-related peptide.

**Table 2:** Comparison of dose, efficacy, and safety of some medications that can be used to treat adolescent obesity in Brazil

Drug	Status	Dose	Efficacy	95% CI	Common adverse events
Liraglutide ( 35 )	Approved for the treatment of adolescent with obesity and obesity or overweight in adults with associated comorbidity.	3.0 mg/day/subcutaneous (titration * )	1,39 Kg/m^2^ BMI reduction	−1.08 to −1.70	Nausea, diarrhea, constipation, vomiting, headache, dyspepsia, fatigue, dizziness, abdominal pain, slight increase in lipase without pancreatitis.
Orlistat ( 31 )	Approved for the treatment of obesity or overweight in adults with associated comorbidity.	360 mg/day	0.79 Kg/m^2^ BMI reduction	−1.08 to −0.51	Oily spotting, flatus with discharge, fecal urgency, fatty/oily stool, increased defecation, fecal incontinence.
Sibutramine ( 31 )	Approved for the treatment of obesity or overweight in adults with associated comorbidity.	10-15 mg/day	1.70 Kg/m^2^ BMI reduction	−2.89 to −0.51	Tachycardia, hypertension, palpitations, insomnia, anxiety, nervousness, depression, diaphoresis.
Metformin ( 31 )	Approved for children over 10 years with DM2.	1,000-2,000 mg/day	1.35 Kg/m^2^ BMI reduction	−2.00 to −0.69	Nausea, flatulence, bloating, diarrhea; usually resolves.
Topiramate ( 47 )	Approved for children and adult epilepsy treatment and adult migraine prophylaxis.	25-100 mg/day	Controversial data	–	Cognitive dysfunction, kidney stones, metabolic acidosis; teratogenic: adolescents must be counseled against pregnancy because of decrease in efficacy of oral contraceptives

* titration (weekly): 0.6, 1.2, 1.8, 2.4 up to 3.0 mg

Abbreviations: BMI, body mass index; CI, confidence interval; DM2, type 2 diabetes mellitus.

Other medications not approved and/or available in Brazil to treat obesity in adolescents are being tested or have scientific evidence of efficacy and safety, such as metformin, topiramate, and exenatide ( 26 , 28 ). Lisdexamfetamine is an FDA-approved drug for the treatment of attention deficit hyperactivity disorder (ADHD) in adults and children (above 6 yrs) and binge-eating disorder in adults ( 28 , 29 ). Semaglutide, which is currently being tested in phase III trials for adults with obesity, is also an option that cannot be disregarded for the treatment of obesity in adolescents in the future since it has a mechanism of action similar to liraglutide and has already shown weight loss benefits in adults with type 2 diabetes ( 29 ). There is an expectation that the combination of phentermine and topiramate will be approved in the next 5 yrs for treating adolescents with obesity. This combination is being evaluated for safety and efficacy in a clinical trial that was launched in 2019. Phentermine and topiramate did not receive approval by the European Medicines Agency (EMA) for the treatment of adults with obesity, and phentermine is not available in Brazil, but there is an ongoing pediatric trial that intends to obtain FDA approval ( 29 ).

One of the main points of this discussion is that there is an urgent need for more clinical trials, and greater efforts must be applied to regulatory agencies for the approval of more anti-obesity medications to treat adolescent obesity. Some medications approved for adults have limited data on safety, efficacy, and follow-up for pediatric patients with obesity. However, the consequences and risks of obesity may outweigh the potentially unknown risks of medications in these patients and must be tested urgently ( 28 , 30 ).

The success of pharmacotherapy, according to the Cochrane Database of Systematic Reviews, is achieved when there is a 5% to 10% decrease in BMI compared to the baseline value ( 31 ). However, it is important to emphasize that adolescents with obesity should not be submitted to pharmacotherapy as a stand-alone approach: all medications must be considered an adjunct to behavioral therapy and lifestyle changes ( 25 ).

### Liraglutide

Liraglutide is an FDA-, EMA- and ANVISA-approved medication for weight management in adults with obesity or overweight who have at least one weight-related coexisting condition ( 32 , 33 ). Recently, liraglutide was approved by the Anvisa and FDA for the treatment of obesity in adolescents aged 12-18 yrs. Brazil was the first country in the world to obtain this regulatory approval in August 2020 ( 26 ).

The mechanism of action of liraglutide has been completely elucidated ( 34 ) ( Table 1 ), and the efficacy and safety for treating adolescents with obesity have been shown in a randomized, phase 3 clinical trial. In this study, 251 adolescents (12-18 yrs) with obesity and a poor response to therapy based on lifestyle changes were randomized to receive up to 3.0 mg of liraglutide subcutaneously once daily or placebo. A total of 103 participants (82.4%) in the liraglutide group and 124 participants (98.4%) in the placebo group reached the maximum dose of the medication. After 56 weeks, a reduction of at least 5% in BMI was seen in 43.3% versus 18.7% of patients who used liraglutide or placebo, respectively. Furthermore, a reduction in BMI of at least 10% was observed in 26.1% of participants in the liraglutide group and 8.1% of participants in the placebo group ( 35 ). The main adverse events reported in the liraglutide group were associated with the gastrointestinal tract, and serious adverse events were rare in both groups ( 35 ). The efficacy, safety, and tolerability profile of liraglutide in this study were similar to that of studies conducted in adults and in previous studies conducted in a smaller population of adolescents as well as in a population of Indian adolescents ( 36 - 38 ). It is important to highlight that liraglutide, at a dose of 3.0 mg/day, has been shown to be safe in regard to neuropsychiatric and cardiovascular outcomes. There were no differences in adverse events of suicidal ideation or behavior reported by patients with obesity treated with liraglutide or placebo ( 35 , 39 ). Cardiovascular safety is also well established, and patients with obesity and DM2 treated with liraglutide did not present higher risk of death from cardiovascular events than those treated with placebo ( 40 ).

### Sibutramine

Sibutramine is approved in Brazil for weight loss and maintenance of weight loss in adults with obesity ( 27 ).

A double-blind, placebo-controlled study with a crossover design was conducted in Brazil and concluded that compared to placebo, sibutramine induced significantly more weight loss in adolescents (10-19 yrs) with obesity. More specifically, 46% and 75% of patients lost 10% of their initial weight in placebo group and sibutramine group, respectively. The patients decreased an average of 1.61 kg and 0.24 kg/m^2^ in placebo group versus 4.47 kg and 2.38 kg/m^2^ (p < 0.001) in the sibutramine group. The most frequent adverse events were headache and diarrhea in the placebo group (4.9%) and headache and constipation in the sibutramine group (13.4%). However, the weight behavior was different according to the timing of sibutramine introduction. Patients who received sibutramine first and started receiving a placebo six months later were unable to maintain weight loss. On the other hand, those who received a placebo first and started receiving sibutramine six months later continued to lose weight. This evidence shows that the introduction of sibutramine is more effective when adherence to lifestyle changes begins to fail ( 41 ). Another Brazilian study showed that adolescents treated with sibutramine lost an average of 10.3 kg (+/-6.6), while in the placebo group they lost 2.4 kg (+/-2.5; p < 0.00). However, the decrease in BMI in the sibutramine group (3.6 +/-2.5 kg/m^2^) compared to placebo (0.9 +/-0.9 kg/m^2^; p < 0.001) was more pronounced than that observed in the previous study. Furthermore, no participant withdrew from the trial because of adverse events, and no difference in blood pressure or heart rate was noted between groups ( 42 ). The Cochrane Database of Systematic Reviews evaluated 568 adolescents with obesity from 5 clinical studies and noted that treatment with sibutramine reduced BMI by 1.70 kg/m^2^ ( 31 ).

The cardiovascular effects of sibutramine in adolescents with obesity are similar to those reported in adults. A clinical trial showed that tachycardia was reported in 13 versus 6% of adolescents treated with sibutramine or placebo, respectively. Furthermore, there were no statistically significant differences in blood pressure between adolescents with obesity treated with sibutramine or placebo. In conclusion, sibutramine treatment seems to have minimal cardiovascular effects and is well tolerated in adolescents ( 43 ).

### Orlistat

Orlistat is FDA approved for long-term use for the treatment of obesity in adolescents (≥12 yrs) but is not approved by EMA ( 29 ). In Brazil, this medication is approved for weight loss and maintenance of weight loss in adults with obesity ( 27 ).

The Cochrane Database of Systematic Reviews evaluated 773 adolescents with obesity from 3 clinical studies and noted that treatment with orlistat reduced BMI by 0.79 kg/m^2^ ( 31 ). However, the main randomized, placebo-controlled, double-blind clinical trial that determines the efficacy and safety of orlistat in weight management among 539 adolescents with obesity (12-16 yrs) was conducted at 32 centers in the USA and Canada. The placebo-subtracted BMI reduction at 52 weeks was 0.86 for orlistat. The dropout rate in this study was similar between the groups: 36% versus 35% in the control group and the orlistat group, respectively. The most common adverse events in the orlistat group were gastrointestinal-related, with most reports indicating adverse events of mild to moderate intensity. These adverse events included steatorrhea, fecal urgency, flatus with oily spotting, abdominal pain, and possible contributions to vitamin D deficiency. Therefore, the clinical use of orlistat is limited because of the modest efficacy (approximately 3% BMI reduction over 12 months) and adverse events ( 44 ).

### Metformin

Metformin is an FDA-approved drug for DM2 (≥10 yrs) that is used without labeling for polycystic ovarian syndrome, insulin resistance, prediabetes, metabolic syndrome, antipsychotic medication-induced weight gain, and stress/emotional eating. Metformin is prescribed as a first-line medication in patients with insulin resistance, prediabetes, or metabolic syndrome and has minimal safety concerns and high tolerability ( 29 ). Due to the lack of options, this medication has been used in Brazil, also without labeling, for the treatment of adolescents with obesity ( 27 ).

A systematic review and meta-analysis of randomized clinical trials evaluated the efficacy of metformin in 2,199 children and adolescents with obesity from 38 studies and found a reduction in BMI after therapy compared to placebo. The weighted mean difference between groups was −1.07 kg/m^2^ (95% CI, −1.43 to −0.72) ( 45 ). A randomized, double-blind, placebo-controlled clinical trial sought to determine whether oral metformin treatment reduces BMI z-score, cardiovascular risk, and inflammatory biomarkers in children with obesity according to pubertal stage and sex. In this study, metformin was effective in reducing the BMI z-score and improved inflammatory and cardiovascular-related obesity parameters in prepubertal (-0.8 in metformin group versus −0.6 in placebo group; p = 0.04) but not in pubertal children (-0.4 in metformin group versus −0.2 in placebo group; p = 0.19) after six months of treatment ( 46 ). In general, metformin is well tolerated by adolescents with obesity, and clinical studies have shown low discontinuity rates due to serious adverse events ( 29 , 46 ).

### Topiramate

Topiramate is approved in the USA for epilepsy treatment and migraine prophylaxis (≥12 yrs) ( 28 ). In Brazil, topiramate is an Anvisa-approved drug for epilepsy treatment in adults and children and migraine prophylaxis in adults. However, even without labeling, topiramate has been prescribed for adolescents with obesity ( 27 ).

Few studies have evaluated the use of topiramate in adolescents with obesity. In a retrospective chart review, adolescents with severe obesity were treated with topiramate along with lifestyle changes for six months; there was a significant decrease in BMI. The results of this retrospective chart review showed a 4.9% BMI reduction (95% CI: −7.1 to −2.8, p < 0.001). The most common adverse events were neurological in nature, including paresthesias, fatigue, dizziness, and memory difficulties ( 47 ). Additionally, the literature review discussed the fact that topiramate is effective in treating obesity, especially in patients with a high BMI at baseline. Therefore, this may be a good choice for patients with obesity and seizures ( 48 ).

### Bariatric surgery

Bariatric surgery is effective in treating adolescents with obesity. Only adolescents with severe obesity (BMI > 35 kg/m^2^ with life-threatening comorbidities) should be considered for this procedure. The Brazilian Ministry of Health authorized bariatric surgery in patients older than 16 yrs in 2013 ( 49 ). The long-term outcomes of surgery are not well established, as clinical trials that validate the long-term efficacy and safety of bariatric surgery for adolescents with severe obesity are scarce. The gold standard for the surgical management of severe obesity in adults and adolescents is Roux-en-Y gastric bypass (RYGB). However, laparoscopic sleeve gastrectomy (LSG) is the most common bariatric surgery performed currently in adolescents ( 50 , 51 ).

A prospective clinical study followed adolescents with obesity (13-21 yrs) who underwent RYGB for 8 yrs. The results showed a 29.2% reduction in BMI 2 yrs postsurgery. Mild anemia, hyperparathyroidism, and low levels of vitamin B12 were the main adverse events ( 52 ). When compared to adults, adolescents with obesity submitted to RYGB showed a similar decrease in BMI after 5 yrs of surgery (26% in adults *versus* 29% in adolescents) ( 53 ).

In 2010, São Paulo University Medical School tested a new technique of bariatric surgery, the Santoro III procedure (sleeve gastrectomy with entero-omentectomy and partial gastroileal derivation). The results of 10 adolescents with obesity at follow-up 1 yr after the Santoro III technique showed a decrease of 19.4 kg/m^2^ in BMI ( 54 ). A retrospective analysis of clinical and laboratory data from 22 adolescents with obesity (14-19 yrs) submitted to LSG showed an average BMI reduction of 12.3 kg/m^2^ in the first 12 months postsurgery. These data indicated that LSG is a safe and efficient procedure to treat adolescents with obesity ( 55 ). The 5-yr postbariatric follow-up showed that weight regain can occur in adolescents who underwent surgery. However, the data showed that this is more common in patients who lost weight without surgical treatment ( 56 ).

### Weight loss benefits

Weight loss decreases or eliminates the comorbidities associated with obesity. The risk of developing DM2 or CVD decrease, but the improvements are not as dramatic as those seen in adults because adolescents with obesity do not normally develop a clinically significant disease. HDL cholesterol, triglyceride levels and insulin resistance also improved after a 5%-10% BMI decrease was achieved. In adolescents with obesity and DM2, bariatric surgery has been associated with remission of DM2 and marked improvements in quality of life ( 57 ).

Furthermore, adolescents with obesity are 50% more likely to develop anxiety and depression than their nonobese healthy peers ( 58 ). However, the depression rate decreases when adolescents lose weight. A systematic review with meta-analysis surprisingly showed that the depression rates improved significantly in the 40 clinical studies evaluated. The other two studies concluded that adolescents who completed the study had significantly lower depression rates than participants who dropped out before the end of the study ( 59 ).

## CONCLUSION

Adolescence is a phase of human development associated with physical, psychological, and social changes. Over the past 40 yrs, the lifestyles of these adolescents have drastically changed, and obesity has become a globally prevalent health problem. The first treatment strategy for adolescents with obesity should be lifestyle change. However, when this strategy is not successful alone, pharmacotherapy can be considered a treatment option. Observational and interventional studies have shown that lifestyle changes combined with anti-obesity drugs can help to reduce the patient's BMI by 5%-10%, and this change has been associated with improvements in cardiovascular and metabolic risk factors. The main problem with this treatment modality is that anti-obesity medications approved for adolescents are limited. Long-term adverse events are the main concerns about the indication of pharmacotherapy for adolescents. Nonetheless, the problems arising from medication can be small when compared to the problems arising from comorbidities associated with early obesity. In other words, there is a clear cost/benefit advantage in this case.

Another difficulty associated with pharmacotherapy is prejudice since obesity is not viewed by parents and patients as a serious and chronic disease that needs long-term treatment. It is important to note that effective and early treatment of obesity can prevent the appearance of serious comorbidities in the future. For more severe cases of obesity, bariatric surgery can be indicated. After reaching the ideal weight, adolescents need to continue medical monitoring for a while, regardless of the strategy used, to avoid weight regain following weight loss. Various compensatory mechanisms (neurologic, hormonal, and behavioral readaptations) have been elucidated, and through these mechanisms, the body may oppose new weight loss, and this compensation may result in weight regain back to the obese baseline. Therefore, after withdrawal from treatment, the weight can be gained back. All efforts must be made to improve the weight of these patients with obesity, which improves their quality of life and health.
